# Measuring infertility in populations: constructing a standard definition for use with demographic and reproductive health surveys

**DOI:** 10.1186/1478-7954-10-17

**Published:** 2012-08-31

**Authors:** Maya N Mascarenhas, Hoiwan Cheung, Colin D Mathers, Gretchen A Stevens

**Affiliations:** 1 Department of Epidemiology and Biostatistics, University of California, San Francisco, USA; 2 The Geisel School of Medicine, Dartmouth University, Hanover, USA; 3 Department of Health Statistics and Informatics, World Health Organization, Geneva, Switzerland

**Keywords:** Infertility, Demographic and health surveys, Population health, Prevalence

## Abstract

**Background:**

Infertility is a significant disability, yet there are no reliable estimates of its global prevalence. Studies on infertility prevalence define the condition inconsistently, rendering the comparison of studies or quantitative summaries of the literature difficult. This study analyzed key components of infertility to develop a definition that can be consistently applied to globally available household survey data.

**Methods:**

We proposed a standard definition of infertility and used it to generate prevalence estimates using 53 Demographic and Health Surveys (DHS). The analysis was restricted to the subset of DHS that contained detailed fertility information collected through the reproductive health calendar. We performed sensitivity analyses for key components of the definition and used these to inform our recommendations for each element of the definition.

**Results:**

Exposure type (couple status, contraceptive use, and intent), exposure time, and outcomes were key elements of the definition that we proposed. Our definition produced estimates that ranged from 0.6% to 3.4% for primary infertility and 8.7% to 32.6% for secondary infertility. Our sensitivity analyses showed that using an exposure measure of five years is less likely to misclassify fertile unions as infertile. Additionally, using a current, rather than continuous, measure of contraceptive use over five years resulted in a median relative error in secondary infertility of 20.7% (interquartile range of relative error [IQR]: 12.6%-26.9%), while not incorporating intent produced a corresponding error in secondary infertility of 58.2% (IQR: 44.3%-67.9%).

**Conclusions:**

In order to estimate the global burden of infertility, prevalence estimates using a consistent definition need to be generated. Our analysis provided a recommended definition that could be applied to widely available global household data. We also summarized potential biases that should be considered when making estimates of infertility prevalence using household survey data.

## Background

Infertility can be a devastating condition for couples who want to have children. Infertility affects men and woman alike, as both genders report associated psychological distress, depression, and low self-esteem [1,2]. In many cultures, the social repercussions of infertility compound the individual impact. Infertility has been observed to result in divorce, loss of economic resources, and even the annulment of rights to burial grounds [3]. Although the global health community has paid considerable attention to fertility, the importance of simultaneously addressing infertility cannot be discounted. Understanding the magnitude and distribution of infertility is key to creating evidence-based policies and reinforcing efforts to reduce the burden of this condition.

A global picture of infertility is not available partly due to the difficulty in defining the condition. In the literature, infertility is used synonymously with sterility, infecundity, childlessness, and subfertility. These terms are used both interchangeably and inconsistently; an explicit detailing of each component of the definition is needed to clarify what is being measured. Gurunath et al. highlighted this lack of consistency in definitions through their systematic review of literature on prevalence studies measuring infertility [4]. The review detailed the inconsistent specification and incomplete documentation of each of four components of the infertility definition – age range, exposure type, exposure time, and outcome.

This inconsistency partly stems from the variety of disciplines that generate infertility measurements. As shown in Table 1, the infertility definitions released by institutions that set guidelines for researchers have not agreed upon a standard definition. The largest disparity lies between the clinical and the demographic definitions. The clinical definitions are oriented toward the early detection of infertility in individual patients with the aim of starting treatment, if necessary, as early as possible. The demographic definition, on the other hand, attempts to measure infertility on a population level, relying on widely applied household surveys rather than sparse data from clinical visits. The clinical definition is important for understanding infertility on an individual level, while the population measures produced by the demographic definition are important inputs to understanding the magnitude, distribution, and underlying trends of infertility at a population level.

**Table 1:** Definitions of infertility found in the literature

**Reference**	**Definition**
International Committee for Monitoring Technology and World Health Organization, 2009 Revised Glossary on ART Terminology [15]	Infertility (clinical definition) is a disease of the reproductive system defined by the failure to achieve a clinical pregnancy after 12 or more months of regular unprotected sexual intercourse.
American Society for Reproductive Medicine, 2008 Definitions of infertility and recurrent pregnancy loss [16]	Infertility is a disease, defined by the failure to achieve a successful pregnancy after 12 months or more of regular unprotected intercourse. Earlier evaluation and treatment may be justified based on medical history and physical findings and is warranted after six months for women over age 35 years.
National Institute for Health and Clinical Excellence guideline 2004 [17]	Infertility should be defined as the failure to conceive after regular unprotected sexual intercourse after two years in the absence of a known reproductive pathology.
World Health Organization, 2001 Reproductive Health Indicators for Global Monitoring [9]	Percentage of women of reproductive age (15–49) at risk of pregnancy (not pregnant, sexually active, noncontracepting, and nonlactating) who report trying for a pregnancy for two years or more.
World Health Organization, 1985 Manual for the investigation and diagnosis of the infertile couple [18]	Infertility, primary: The woman has never conceived despite cohabitation, exposure to pregnancy, and the wish to become pregnant for at least 12 months. Infertility, secondary: The woman has previously conceived but is subsequently unable to conceive despite cohabitation, exposure to pregnancy, and the wish to become pregnant for at least 12 months. If the woman has breastfed a previous infant, then exposure to pregnancy should be calculated from the onset of regular menstruation following delivery.
Demographic definition, 1985 The dictionary of demography [19]	The inability to produce a live birth. The term usually refers to women, but men or couples can be the focus of attention. Used without qualification, sterility implies irreversibility, but the term temporary sterility is sometimes used. A distinction is made between primary sterility where a woman has never been able to have a child, and secondary sterility, which occurs after the birth of at least one offspring.
World Health Organization, 1975 The Epidemiology of Infertility – Report of a WHO Scientific Group [8]	Primary infertility: The woman has never conceived despite cohabitation and exposure to pregnancy for at least two years. Secondary infertility: The woman has previously conceived but is subsequently unable to conceive despite cohabitation and exposure to pregnancy for a period of two years; if the woman has breastfed a previous infant, then exposure to pregnancy should be calculated from the end of the period of lactational amenorrhea.

In this paper, we developed a demographic definition of infertility that may be applied to a range of publicly available national household surveys for the purpose of generating a consistent measure of the prevalence of infertility. All infertility measurements – clinical, epidemiologic, and demographic – incorporate some time-dependent measurement of exposure, i.e., a couple is classified as infertile if they have tried unsuccessfully to become pregnant or give birth for more than a minimum length of time. Most demographic household surveys do not directly collect information on the length of time that a couple has been trying to conceive. However, exposure can be inferred from a woman’s couple status, contraceptive use, and desire for a child, all measured over a defined period of time. These indirect measures are available across most demographic and reproductive health surveys. The level of detail available for each measure can vary by survey and may lead to biases. In order to account for potential sources of measurement error in our surveys, we evaluated the effects of varying individual components of our infertility definition and made recommendations accordingly.

Previous studies have evaluated demographic definitions of infertility using simulation analyses, with the aim of constructing a definition for high-fertility settings [5,6]. Larsen and Menkin [5] used simulation analyses to test the sensitivity of different measures of infertility to survey sample size, age distribution of sterility, age at marriage, and fecundity. They found that the five-year “subsequently infertile” measure, defined as the percent of continuously married couples who do not bear a child during a five-year observation period, was most robust to the factors considered in their sensitivity analyses. Larsen [6] extended her previous work to analyze the error arising from contraceptive use through microsimulations. She found that contraceptive use could be ignored if fewer than 6% of women were users. Larsen [7] collected primary data in Northern Tanzania to compare the World Health Organization’s (WHO) epidemiological definition of infertility, trying without success to bear a child for two years or more [8,9], to one derived from data commonly available in demographic and fertility surveys. She found that the five-year “subsequently infertile” measure was appropriate only when it was limited to women who had expressed a desire to have a child in addition to being continuously married with no successful birth during the exposure period.

We based our definition of infertility on the “subsequently infertile” estimator proposed by Larsen and colleagues; however, we extended their previous work in this paper for two reasons. First, the proportion of women of reproductive age using contraception has increased around the world, ranging from 24% in Africa to 43% to 80% in other regions [10]. This significant change necessitates the use of a definition that is robust to contraceptive use greater than 6%. Second, more detailed exposure measurements are now available from many nationally representative surveys. Specifically, the Demographic Health Survey (DHS) has now collected information on couple status and contraceptive use over a five-year period in a number of countries around the world.

Our proposed definition may be used to estimate country, regional, and global prevalence of infertility and its trends from the large pool of available demographic household surveys. It is relevant across diverse populations with different rates of marriage, contraceptive use, and childbearing practices. By presenting a consistent and widely applicable definition, as well as quantifying the biases that arise from using common variations of this definition, this paper provides a valuable framework for future estimates of the global burden of infertility.

## Methods

**Figure 1:**
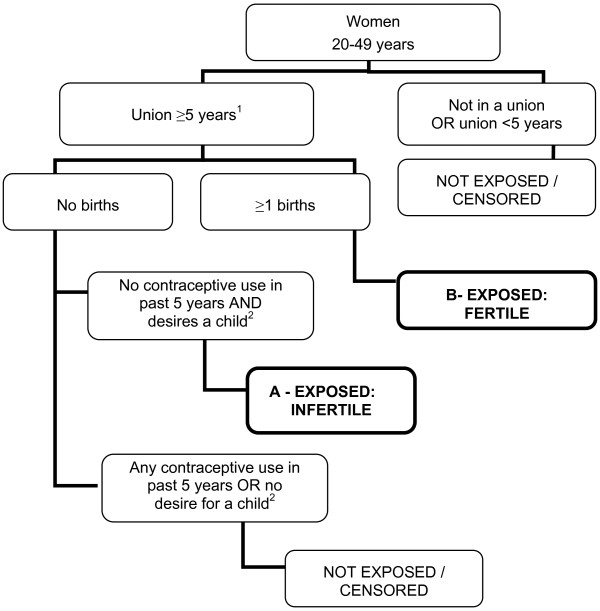
**Primary infertility, women aged 20 to 49 years using a five-year exposure period.** Primary infertility prevalence is calculated as the number of infertile women (**A**) divided by the number of women who are both infertile and fertile (**A** and **B**). 1: Union is defined as marriage or cohabitation. 2: Desire for a child is defined as wanting a child, undecided, or declared infecund.

**Figure 2:**
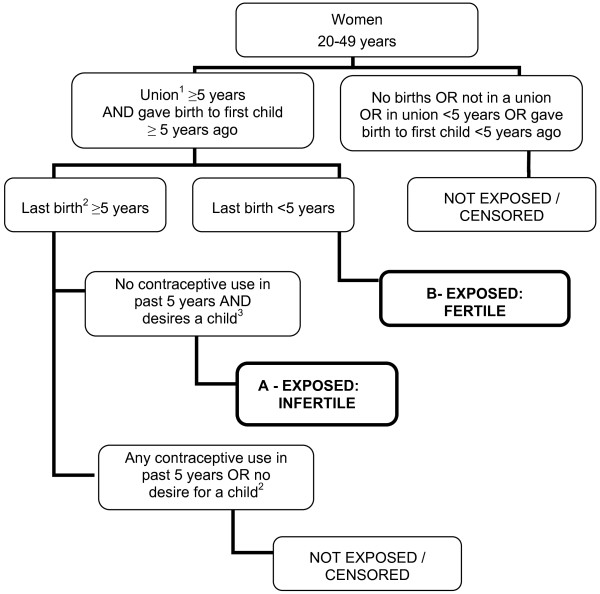
**Secondary infertility, women aged 20 to 49 years using a five-year exposure period.** Secondary infertility prevalence is calculated as the number of infertile women (**A**) divided by the number of women who are both infertile and fertile (**A** and **B**). 1: Union is defined as marriage or cohabitation. 2: Last birth refers to the most recent birth after the first child. 3: Desire for a child is defined as wanting a child, undecided, or declared infecund.

Few papers in the literature present estimates of infertility prevalence using clear and complete definitions. Household surveys provide a robust alternative to published studies for obtaining comparable prevalence estimates. We propose a standard definition of primary and secondary infertility that can be applied to these surveys, as detailed below:

1) Primary infertility is defined as the absence of a live birth for couples that have been in a union for at least five years, during which neither partner used contraception, and where the female partner expresses a desire for a child. The prevalence of primary infertility is calculated as the number of woman in an infertile union divided by the combined number of women in fertile and infertile unions. Women in a fertile union have had at least one live birth and have been in a union for at least five years at the time of the survey (Figure 1).

2) Secondary infertility is defined as the absence of a live birth for couples that have been in a union for at least five years since the female partner’s last live birth, during which neither partner used contraception, and where the female partner expresses a desire for a future child. The prevalence of secondary infertility is calculated as the number of women in an infertile union divided by the combined number of women in infertile and fertile unions. Women in a fertile union have had at least one live birth in the past five years and, at the time of the survey, have been in a union for at least five years following their first birth (Figure 2).

We used DHS data to explore how varying key components of each definition affected prevalence estimates. The components we assessed were exposure time, exposure type (couple status, contraceptive use, and desire for a child), and outcome. To determine the effect of varying each component with respect to our proposed definition, we assessed the associated median relative errors and the interquartile range of relative errors, measures which are not unduly affected by surveys with very small sample sizes.

Every standard DHS questionnaire collects data from women of reproductive age on couple status, birth history, contraceptive use, and desire to have a child. A subset of DHS include the reproductive health calendar, a tool used to collect detailed information on couple status, contraceptive use, and birth history for each month spanning the five years prior to the survey. In order to compare the prevalence estimates generated using the detailed measures from the reproductive health calendar to those generated using standard survey questions, we restricted our analyses to the subset of surveys that included both the standard questionnaire and the reproductive health calendar.

Infertility measures like the one proposed here reflect etiologies that can be attributed to the male partner, the female partner, or both partners. Because the DHS interview women of reproductive age, our analysis was indexed by the age of the women in each couple, and we refer to women and couples interchangeably in the text. We excluded women who had been exposed for less than the minimum exposure period from the analysis. Prevalence estimates were generated for six age groups (20–24, 25–29, 30–34, 35–39, 40–44, and 45–49) indexed by the age of the female survey respondent at the time of the survey. We calculated prevalence using sample weights to account for complex survey design. We generated age-standardized estimates using the WHO reference population [11].

## Results

We analyzed 53 surveys representing 26 countries and spanning the years 1990 to 2008 (Table 2). Using the proposed definitions, we found that the age-standardized prevalence of primary infertility ranged from 0.6% (95% confidence interval [CI]: 0.4%, 0.8%) in Peru in 1992, to 3.4% (95% CI: 2.7%, 4.1%) in Morocco in 1992. The age-standardized prevalence of secondary infertility ranged from 8.7% (95% CI: 7.0%, 10.7%) in Zimbabwe in 2005 to 32.6% (95% CI: 27.8%, 37.8%) in Armenia in 2000.

**Table 2:** Age-standardized primary and secondary infertility prevalence estimates, women aged 20-49 years as calculated using selected Demographic and Health Surveys

**Country**	**Year**	**Primary**		**Secondary**		**Survey size**
		**Prevalence (%)**	**Sample size**	**Prevalence (%)**	**Sample size**	
Armenia	2000	1.4 (1.0, 2.0)	3584	29.2 (24.8, 34.0)	745	5262
Armenia	2005	1.3 (0.9, 1.9)	3424	32.6 (27.8, 37.8)	647	5430
Bangladesh	1993	1.7 (1.4, 2.1)	7030	18.5 (16.4, 20.8)	3758	8225
Bangladesh	1996	1.5 (1.2, 1.9)	6497	21.8 (19.8, 24.0)	3240	7709
Bangladesh	1999	1.8 (1.5, 2.1)	7406	18.3 (16.3, 20.5)	3377	8922
Bangladesh	2004	1.7 (1.4, 2.0)	8114	20.5 (18.4, 22.8)	3610	9737
Bolivia	1994	1.0 (0.7, 1.4)	4099	9.5 (8.1, 11.2)	2525	6780
Brazil	1991	1.5 (1.1, 2.1)	2516	12.4 (9.4, 16.1)	1122	4805
Brazil	1996	1.6 (1.3, 2.1)	5668	16.7 (14.3, 19.4)	1825	10075
Colombia	1990	1.6 (1.1, 2.4)	3234	12.9 (9.0, 18.0)	1273	6835
Colombia	1995	1.4 (1.0, 1.9)	4326	13.2 (11.0, 15.8)	1655	8969
Colombia	2000	1.5 (1.1, 2.1)	4295	15.9 (13.2, 19.0)	1433	9319
Colombia	2005	1.5 (1.2, 1.8)	14159	15.9 (14.1, 17.9)	4591	31047
Democratic Republic of the Congo	2002	1.7 (1.4, 2.1)	10189	23.1 (20.6, 25.7)	3670	18576
Dominican Republic	1991	2.2 (1.6, 3.1)	2806	16.2 (12.1, 21.3)	1183	5596
Dominican Republic	1996	2.4 (1.8, 3.1)	3539	19.2 (15.9, 23.0)	1413	6584
Dominican Republic	1999	1.0 (0.4, 2.4)	532	23.7 (15.3, 34.8)	190	1018
Egypt	1995	2.8 (2.4, 3.2)	10817	13.2 (11.8, 14.8)	5666	14075
Egypt	2000	2.7 (2.3, 3.1)	11148	13.1 (11.7, 14.8)	5281	14978
Egypt	2005	2.6 (2.3, 3.0)	13719	15.0 (13.6, 16.6)	6255	18616
Egypt	2008	3.1 (2.7, 3.5)	11495	14.7 (13.2, 16.4)	4851	15891
Ethiopia	2005	1.3 (0.9, 1.7)	6617	11.7 (10.3, 13.1)	4883	10818
Guatemala	1995	1.0 (0.7, 1.4)	6384	16.6 (14.9, 18.4)	4379	9454
India	2005	2.7 (2.5, 2.9)	71095	24.6 (23.3, 26.0)	22740	100430
Indonesia	1991	2.6 (2.2, 3.1)	16961	18.0 (16.6, 19.6)	8289	21910
Indonesia	1994	2.1 (1.8, 2.5)	21059	21.2 (19.3, 23.3)	10080	27085
Indonesia	1997	2.6 (2.2, 3.0)	21295	20.4 (18.6, 22.3)	9509	27707
Indonesia	2002	2.6 (2.2, 3.0)	22087	18.8 (16.9, 20.8)	8847	28559
Indonesia	2007	2.1 (1.8, 2.5)	24616	17.7 (16.2, 19.3)	10479	31981
Jordan	1997	2.5 (2.0, 3.1)	4087	9.8 (8.1, 11.7)	2591	5342
Kazakhstan	1999	1.1 (0.7, 1.7)	2410	25.9 (20.5, 32.2)	609	4022
Kenya	1998	1.0 (0.7, 1.4)	3584	15.6 (13.3, 18.2)	2242	6029
Kenya	2003	1.0 (0.7, 1.5)	3508	11.6 (9.7, 13.8)	2241	6375
Malawi	2004	1.0 (0.7, 1.3)	5624	10.1 (8.8, 11.7)	4090	9291
Morocco	1992	3.4 (2.7, 4.1)	4059	10.8 (9.3, 12.5)	2568	7111
Nicaragua	1997	1.1 (0.9, 1.5)	6005	12.5 (10.7, 14.6)	3085	10277
Paraguay	1990	1.5 (1.2, 2.0)	2722	15.8 (13.8, 18.1)	1699	4533
Peru	1992	0.6 (0.4, 0.8)	7052	10.8 (9.5, 12.3)	3756	12398
Peru	1996	0.8 (0.6, 1.0)	13715	10.0 (8.9, 11.4)	7272	22897
Peru	2000	1.0 (0.8, 1.3)	12938	10.5 (9.3, 11.9)	5913	22095
Philippines	1993	2.1 (1.7, 2.5)	7190	15.1 (13.7, 16.7)	4102	11890
Philippines	1998	1.8 (1.5, 2.3)	6709	17.0 (15.3, 18.8)	3650	11034
Philippines	2003	2.7 (2.1, 3.3)	6778	17.7 (16.0, 19.5)	3302	10987
Moldova	2005	3.2 (2.4, 4.3)	3894	29.5 (25.0, 34.4)	708	6037
Turkey	1993	2.3 (1.8, 2.8)	4916	18.0 (15.4, 21.0)	1688	6189
Turkey	1998	2.6 (2.1, 3.2)	4644	20.1 (17.5, 22.9)	1661	6813
Turkey	2003	2.3 (1.7, 3.1)	3322	15.9 (12.4, 20.1)	1084	7835
Tanzania	2004	1.8 (1.4, 2.3)	4714	16.5 (14.8, 18.3)	3511	8032
Viet Nam	1997	1.0 (0.7, 1.4)	4300	12.2 (9.5, 15.4)	1478	5551
Viet Nam	2002	0.8 (0.6, 1.2)	4504	13.8 (10.0, 18.7)	1059	5598
Zimbabwe	1994	1.1 (0.8, 1.7)	2650	14.6 (12.5, 17.1)	1813	4642
Zimbabwe	1999	1.6 (1.1, 2.2)	2385	13.9 (11.5, 16.8)	1472	4439
Zimbabwe	2005	1.2 (0.8, 1.6)	3567	8.7 (7.0, 10.7)	2050	6777

95% confidence intervals are shown in parentheses. Sample size refers to the proportion of women who were included in the calculations of primary or secondary infertility (i.e., the denominator in the prevalence calculation), and the survey size refers to the total number of women surveyed.

### Exposure time

Exposure time refers to the minimum time necessary to assess whether a union is infertile. Using the reproductive health calendar, we produced an estimate of infertility that captured monthly couple status and contraceptive use over the exposure period. We compared the use of one- and two-year exposure periods to the five-year exposure period used in our proposed definition.

**Figure 3:**
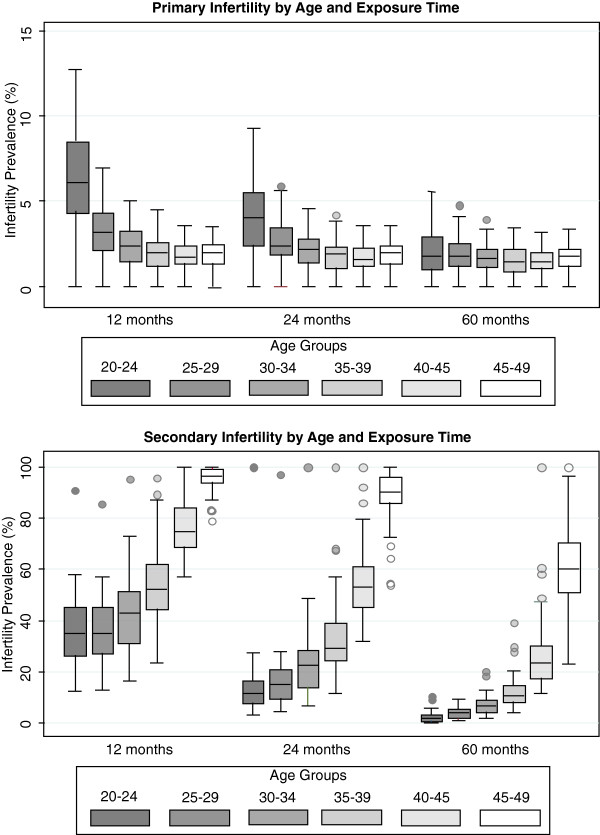
**Boxplots of primary and secondary infertility prevalence by age for 12-, 24-, and 60-month exposure periods, 53 Demographic and Health Surveys.** The boxplots depict the distribution of values for each estimate, showing the range with the whiskers and the interquartile range and median with the box.

We found strong evidence of misclassification when using exposure times of one and two years. Shorter exposure times are more sensitive to identifying women in an infertile union, while a longer exposure time is less likely to misclassify women as infertile who, in the absence of intervention, would have given birth. The prevalence estimates for primary and secondary infertility were inversely related to exposure time (Figure 3). The shorter exposure times of one and two years produced a strong age trend; prevalence estimates of infertility were highest in the youngest age groups and declined as women aged. This pattern of decreasing infertility prevalence in older women is highly implausible.

Because the proposed definition uses birth as an outcome, the exposure period must accommodate gestation time in addition to time to conception. When shorter exposure times are used, pregnant women are more likely to be labeled as being in an infertile union because the exposure period is less likely to be long enough to capture the birth. The percent of women who are classified as infertile but are pregnant at the time of the survey can be used as an indicator of misclassification. Tellingly, this percentage decreases as exposure time increases for both primary and secondary infertility**.** For primary infertility, the median age-standardized percent of infertile women who are pregnant using a five-year exposure time is 3.8% (interquartile range [IQR]: 1.7%-5.3%) compared to 10.8% (IQR: 9.2%-12.6%) using a one-year exposure time; for secondary infertility, the percent increases from 3.9% (IQR: 1.8%-6.6%) to 10.2% (IQR: 7.6%-13.2%).

### Couple status

Marriage and cohabitation are measures found in all standard household surveys. In many countries with high fertility rates, they also provide a good proxy for exposure, particularly when combined with other variables assessing contraceptive use and intent.

In the majority of DHS, the duration of couple status is measured from the time of first union (marriage or cohabitation). As a result, it is impossible to accurately capture a continuous measure of union length for women who have been in multiple unions. Measuring time since first union for women with multiple unions has the potential to overestimate a woman's exposure time to pregnancy and the corresponding prevalence of infertility. Limiting the analysis to women in their first and only union, however, can produce an underestimate of the prevalence if infertile women are more likely to have multiple unions or women with multiple unions are predisposed to contract sexually transmitted diseases that cause infertility. In addition, DHS do not collect information on past temporary separations, for example due to migratory work, which can also result in an overestimate of the exposure time.

The reproductive health calendar collects detailed information on couple status during the five-year period prior to the survey and can be used to assess the biases from using the measurement of time from first, rather than current, marriage. In order to assess the impact of incorporating time since first union into the exposure measure, we examined the effects of including women with multiple unions and of limiting the sample to women in their first and only union. We compared both sets of prevalence estimates to estimates produced by using the reproductive health calendar, which captured a continuous measure of couple status over five years irrespective of whether it was a first or subsequent union. The magnitude of either sets of biases proved to be small. The median relative error in age-standardized prevalence from using time since first marriage for women in their first and only union compared to using all women with a continuous measure of couple status was −4.5% (IQR: -9.9%-0.0%) for primary infertility and −5.3% (IQR: -10.4% - -1.9%) for secondary infertility. The equivalent comparison of time since first marriage and the continuous measure of couple status amongst all women, including those with multiple marriages, produced median relative percent differences of 5.2% (IQR: 0.5%-9.6%) for primary infertility and differences of 4.0% (IQR: 2.1%-9.7%) for secondary infertility.

Although information is not available on past separations during the exposure period, women are asked to report whether their partners are currently living in the same household. We assessed the effect of excluding women whose husbands were living elsewhere at the time of the survey from the infertility measure. We found that the effect was negligible: the median relative error in age-standardized prevalence was 0.6% (IQR: -2.0%-2.2%) for primary infertility and −1.2% (IQR: -3.5% - -0.5%) for secondary infertility.

### Contraceptive use

Unprotected sex is generally necessary for conception and thus serves as a useful proxy for exposure. The reproductive health calendar allowed us to measure the continuous use of contraception over the exposure period; however, most household surveys only collect information on current rather than continuous usage. In order to determine whether current contraceptive use is a sufficient proxy for continuous use over a five-year period, we compared the prevalence estimates generated from current use to those generated using a continuous monthly measure.

Prevalence estimates using a current, rather than continuous, measure of contraceptive use produced estimates biased upwards for primary and secondary infertility. However, the patterns were different for each type of infertility (Table 3). For primary infertility, the median relative error between current and continuous usage was largest in the younger age groups but was small for ages 30 and above (Table 3), producing median age-standardized differences of 5.5% (IQR: 2.7%-14.9%). For secondary infertility, large differences were observed across all age groups, with a median age-standardized difference of 20.7% (IQR: 12.6%-26.9%).

**Table 3:** Relative percent differences from sensitivity analyses of key components of the infertility definition - contraception, intent, and outcome

**Age group**	**20-24**	**25-29**	**30-34**	**35-39**	**40-44**	**45-49**	**Age-standardized**
**Primary infertility**							
Contraception^a^	9.2	5.8	4.3	0.0	0.0	0.0	5.5
	(0.0, 31.9)	(0.0, 19.2)	(0.0, 13.2)	(−0.1, 6.8)	(−0.1, 3.5)	(0.0, 0.0)	(2.7, 14.9)
Intent^b^	0.0	0.0	0.0	2.2	2.1	11.4	4.1
	(−0.1, 0.0)	(0.0, 1.5)	(0.0, 3.3)	(0.0, 6.8)	(0.0, 12.7)	(0.0, 20.3)	(1.7, 7.7)
Outcome^c^	−10.1	−4.6	−1.5	0.0	0.0	0.0	−3.4
	(−16.7, 0.0)	(−9.1, 0.0)	(−6.2, 0.0)	(−1.4, 0.0)	(0.0, 0.0)	(0.0, 0.0)	(−5.2, -1.7)
**Secondary infertility**							
Contraception^a^	129.9	102.1	54.1	23.6	12.0	5.3	20.7
	(73.1, 331.5)	(57.6, 169.6)	(32.4, 80.3)	(12.4, 39.9)	(6.9, 23.8)	(2.4, 10.3)	(12.6, 26.9)
Intent^b^	4.5	29.1	41.3	84.4	104.9	37.9	58.2
	(0.0, 28.2)	(16.8, 34.8)	(27.0, 60.8)	(59.6, 106.8)	(65.9, 131.7)	(20.8, 53.5)	(44.3, 67.9)
Outcome^c^	−16.7	−2.8	−0.8	0.0	0.0	0.0	−0.7
	(−50.0, 0.0)	(−5.9, 0.0)	(−3.0, 0.0)	(−0.5, 0.0)	(0.0, 0.0)	(0.0, 0.0)	(−1.1, -0.3)

a- Current contraception (alternate) compared to continuous contraception (baseline) over the exposure period.

b- Not using the criteria of “desire for a child” variable (alternate) compared to using it to determine a couple's infertility status (baseline).

c- Assigning infertile woman who are pregnant as fertile (alternate) compared to classifying them as infertile (baseline).

Relative percent differences were calculated as the alternate prevalence less the baseline prevalence, divided by the baseline prevalence. The median and the interquartile range across all surveys are shown.

### Intent

The inclusion of a woman’s intent to conceive serves as a proxy for unprotected sexual intercourse, because women who explicitly do not want children are likely to employ protective measures to avoid conception. Women who did not desire a child were excluded from the infertile group, even if they met all other criteria for infertility. Women who did not provide an answer, were undecided, self-identified as infertile, or stated that they wanted a child were assessed according to the remaining criteria for infertility. Excluding women who did not desire a child from the analysis did not notably affect primary infertility estimates, especially in the younger ages (Table 3), producing a median age-standardized relative difference of 4.1% (IQR: 1.7%-7.7%). For secondary infertility, the prevalence differences were larger and produced an overall median age-standardized difference of 58.2% (IQR: 44.3%-67.9%).

### Outcome

Because couples desire children, not simply pregnancies, infertility affects couples regardless of whether the etiology lies in conception or in the progression of the pregnancy. Furthermore, distinguishing between the two is often not possible in household surveys. Births are reported quite accurately in household surveys, while pregnancies that do not result in a birth (planned and spontaneous abortions) are not as reliably reported [12]. Spontaneous abortions are often underreported since mothers may not be aware of their pregnancy status during the first trimester when spontaneous abortions are most likely to occur. Pregnancies that end in planned abortions are also underreported due to social stigma and legal issues.

We used live birth as the outcome variable when assigning infertility to couples. As a result, some women who were pregnant at the time of survey were categorized as being in an infertile union because they had not yet achieved a live birth. This resulted in a potential overestimate of prevalence. We calculated a set of prevalence estimates assuming that women were in a fertile union if they were pregnant at the time of survey. Not all women report they are pregnant during the first trimester so we expect that the proportion of self-reported pregnant women would not fully capture the potential misclassification; however, this effect is partially counteracted by pregnancies that do not result in births. For primary and secondary infertility, differences when using pregnancy as the outcome were only notable for 20- to 29-year-olds (Table 3). This pattern may reflect the fact that woman in these age groups had the highest rates of overall pregnancies.

## Discussion

The literature lacks clear and consistent measures of infertility [4]. Through analyses of the DHS, we made recommendations for a definition of infertility with the goal of informing future estimates of the burden of infertility (Table 4). Our analysis produced a definition that can be applied consistently to a variety of household survey data with information on fertility. We constructed this definition by considering the multiple components of infertility and performing sensitivity analyses on each (Table 3). By using information from the reproductive health calendar, we were able to generate a definition that could assess changing behaviors over a period of up to five years. We compared these to prevalence estimates based on current measures of behavior, which are more commonly found in household surveys.

**Table 4:** Recommendations for defining infertility in analyses of household survey data based on sensitivity analyses of key components of the infertility definition

**Definitional component**	**Recommendation**	
	**Primary infertility**	**Secondary infertility**
**Exposure time**	One- to two-year exposure periods increase misclassification of fertile unions as infertile; a five-year exposure period is recommended.	
**Couple status**	Measuring time since first union as a proxy for couple status results in an acceptable error (< 5%) for prevalence estimates, even for women with multiple unions. Temporary separations have little effect on infertility estimates	
**Contraception**	Current contraception is a sufficient proxy for contraceptive use over the exposure period for women over 30.	Current contraception is not a sufficient proxy for contraceptive use over the exposure period.
**Intent**	Intent has a small influence on prevalence estimates in the surveys analyzed, although this may not be true for high-income settings.	Disregarding intent increases estimates of infertility. Taking into account intent is recommended when measuring the disability of secondary infertility.
**Outcome**	Using reported birth is recommended as it is a more reliable measure than reported pregnancies. When using birth as an outcome, some women classified as infertile are pregnant at the time of the survey. The proportion of infertile women who are pregnant is smaller if longer exposure times are used.	

We found that incorporating a five-year exposure time was important for an accurate measure of infertility. We focused on improving specificity rather than sensitivity because demographic and health surveys tend to produce estimates of infertility that are biased upwards when compared to more targeted infertility surveys [7,13]. Our analyses showed that an exposure measure of five years, rather than one or two years, reduced the likelihood of misclassifying fertile unions as infertile in our estimates; a longer exposure period allows for the time that it takes to conceive and bear a child and prevents unreported temporary separations or periods of abstinence from unduly affecting the infertility measure. Prior analyses have also indicated that prevalence estimates generated using a five-year measure are most similar to the prevalence of permanent sterility [5], which causes the greatest health burden.

Larsen has argued that couples who use contraceptives are more fertile than those who do not, and thus should be classified as fertile [6,7]. However, it is by definition not possible to determine whether contraceptive users would have conceived in the absence of contraceptive use, regardless of the instrument used to determine infertility status. Worldwide, 63% of women of reproductive age currently use contraceptives [10]. Given the high current prevalence of contraceptive use, it is likely that users are more representative of the general population than they were in the high-fertility settings Larsen studied. Thus, we argue that it is no longer possible to assume that all couples using contraceptives are fertile, and a robust definition of infertility should incorporate a measure of contraception over the exposure period.

There are limitations to using household surveys for estimating the prevalence of infertility. Due to the nonspecific nature of household survey questions, we had to construct a definition of infertility that used couple status, contraceptive use, and intent to conceive as proxies for regular unprotected sexual intercourse, typically not measured in household surveys. Assigning infertility to women who expressed intent to conceive served as a proxy for regular, unprotected sexual intercourse, and may correct for underreporting of contraceptive use [7]. Nevertheless, there is a risk that women who are unable to have a child may cope by changing their stated or experienced fertility preferences, which would discount the prevalence of infertility and underestimate its burden [14]. We extended the exposure period to reduce the likelihood of misclassifying fertile couples as infertile. A longer exposure period, however, resulted in a smaller sample and increased the likelihood of recall bias. There is also a risk that infertility can lead to voluntary dissolution of the union in fewer than five years, which would not be captured by our definition. Furthermore, survey questions built around sensitive topics such as childlessness and contraceptive use have the potential to produce responses biased by social norms. While household surveys are an invaluable source of data, surveys that include explicit questions on infertility or contain detailed measures of important components are also needed. Population-based studies that include clinical assessments are a vital input to identifying the causes underlying this condition and the relative contribution of male and female factors.

## Conclusions

The definition presented in this paper can guide the use of household survey data for the measurement of infertility from sources such as the standard DHS, the World Fertility Surveys, the Pan Arab Family and Child Health Surveys, the Centers for Disease Control Reproductive Health Surveys, and the National Survey for Family and Growth Study (United States). Although the above surveys do not all collect detailed information on past reproductive health practices that were used in this paper, we have quantified the potential biases and made corresponding recommendations for how to best use the more commonly collected data found in these surveys (summarized in Table 4). The analysis presented in this paper will allow for a comprehensive global understanding of infertility prevalence using the large number of nationally representative household surveys available. Future analyses can build upon this work by examining the relationship of infertility, calculated using demographic and fertility surveys, with its determinants, including the prevalence of sexually transmitted diseases, female age at first pregnancy, and indicators of nutritional status such as the prevalence of female anemia or underweight. A better understanding of the prevalence and causes of infertility, in turn, will enable informed policy changes regarding prevention and treatment to effectively reduce this global burden.

## Abbreviations

DHS: Demographic and health surveys; IQR: Interquartile range; WHO: World health organization.

## Competing interests

The authors declare that they have no competing interests.

## Authors’ contributions

MNM, GAS, and CDM developed the study content and design. MNM extracted and analyzed survey data. MNM wrote the first draft of the report. MNM, GAS, HC, and CDM all edited and approved the final version. GAS oversaw the research process. All authors read and approved the final manuscript.
